# Branch retinal artery occlusion caught in the act by an optical coherence tomography angiography image: case report

**DOI:** 10.1186/s12886-022-02517-5

**Published:** 2022-07-14

**Authors:** Fabio Scarinci, Andrea Cacciamani, Guido Ripandelli, Mariacristina Parravano

**Affiliations:** IRCCS – Fondazione Bietti, Via Livenza, 3, 00198 Rome, Italy

**Keywords:** Retinal artery occlusion, Spectral domain optical coherence tomography (SD-OCT), Optical coherence tomography angiography (OCTA), Case report

## Abstract

**Background:**

Retinal artery occlusion is a vascular entity caused by the temporary blockage of retinal arterioles.

**Case presentation:**

We present the case of a 57-year-old woman a partial visual loss in the right eye due to a cilioretinal artery occlusion. Ophthalmoscopy revealed a focal area of retinal whitening superior to the optic nerve in the right eye, while the left eye was within the limit.

Retinal imaging, in particular optical coherence tomography angiography (OCTA), showed a capillary drop out of the superficial capillary plexus and the corresponding b-scan showed a round hyporeflective grey dot (optical empty) corresponding to the dark grey spot on the enface view at the level of the retinal whitening area.

**Conclusion:**

Although the images did not allow the differentiation between vasospasm or retinal emboli, the OCTA imaging might help to identify and to caught in the act the specific region causing the retinal impairment. Also, the possible formation of small microcavity should be considered in case with branch retinal artery occlusion. The use of this new imaging technology might help to evaluate the efficacy of the therapy in vivo.

## Background

Retinal artery occlusion is a vascular entity caused by the temporary blockage of retinal arterioles. The occlusion can affect either the central retinal artery or a branch of this vessel. In this case, patients usually present sectoral visual field defect [1].

The vasospastic syndrome might involve the ocular circulation, especially in patients with migraine, and rarely cause a retinal arteriolar vasospasm [2].However, also minor microvasculature thromboembolic events of the capillary network might produce localized ischemic lesions.

In this case, non-invasive imaging modalities including fundus autofluorescence [3] and optical coherence tomography angiography (OCTA) [4, 5] have provided structural insights and also showed, for the first time in vivo, the vascular alteration into this retinal disease.

## Case presentation

A 57-year-old woman presented with visual complaints described as a “foggy area” in the right eye.

There was no history of classic migraine, ocular pain, flashes, recent trauma or visual loss. The past medical history was also negative for any thromboembolic episodes as well as any primary Raynaud’s phenomenon. She was a non-smoker and referred being on a high dietary protein intake in the last few months. Recently, she was diagnosed with high blood pressure and was taking medication as prescribed. Haematological examinations were within the limits excluding the presence of high cholesterol levels.

In both eyes, the optic nerve was normal. There was not relative afferent pupillary defect and full colour vision was within the limits. Her best corrected visual acuity was 20/20 in both eyes. Both of her eyes were quiet with no conjunctival hyperaemia and no anterior chamber inflammation.

Ophthalmoscopy revealed a focal area of retinal whitening superior to the optic nerve consisting with branch retinal artery occlusion. There was no evidence of disc swelling or haemorrhages and examination of her fellow eye was normal. Gentle, prolonged ocular massage was applied.

Optical coherence tomography (OCT) and optical coherence tomography angiography (OCTA) (Heidelberg Engineering, Heidelberg, Germany) images were performed to rule out any other retinal lesions involving the macular area.

**Fig. 1:**
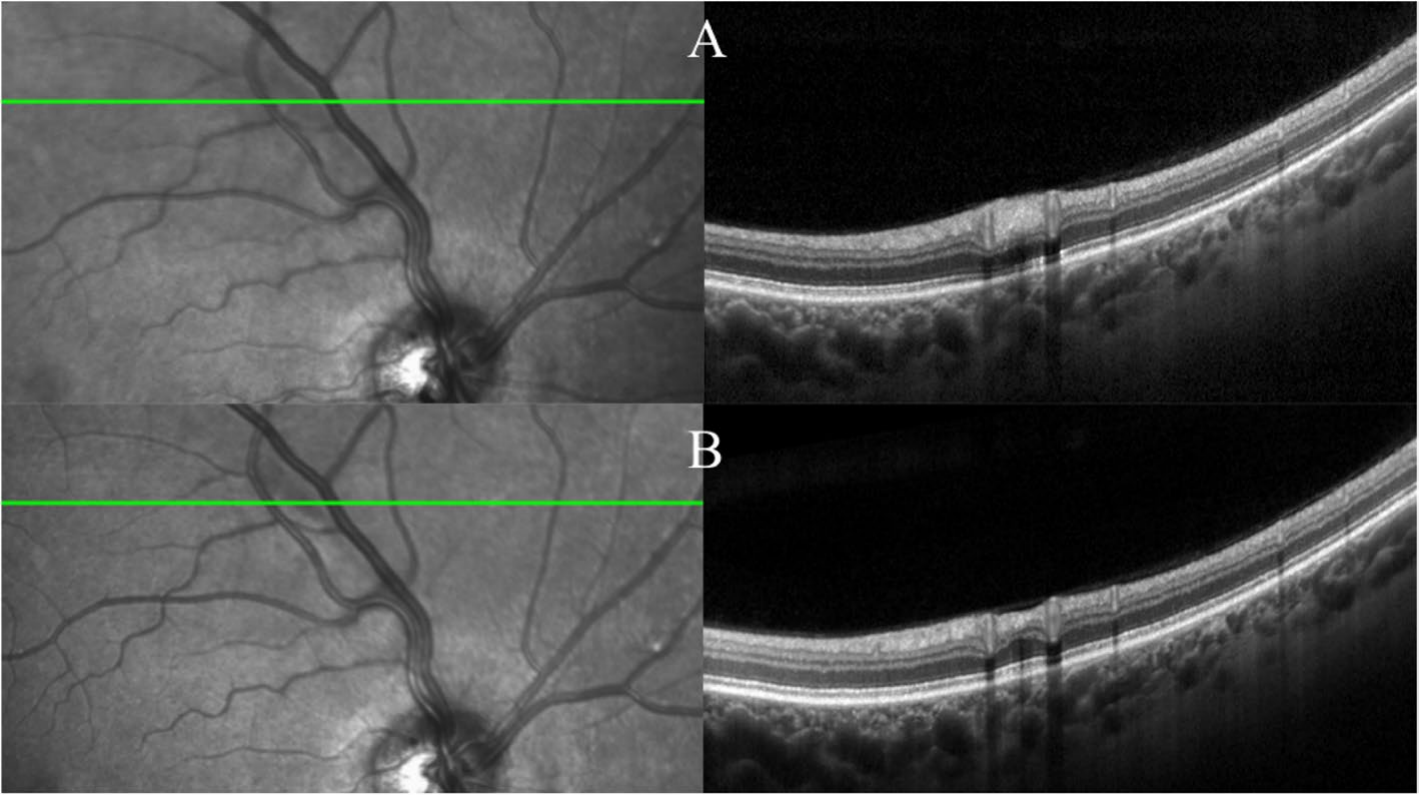
Superiorly to the optic nerve, the ischemic lesion showing corresponding inner nuclear, inner plexiform, ganglion cell, and retinal nerve fibre layer on spectral domain optical coherence tomography (**A**). Four weeks after initial presentation affected layers revealed thinning and the inner nuclear layer is only partially identifiable (**B**)

On presentation, the OCT b-scan shows a normal macular area in both eyes. Conversely, in the corresponding retinal whitening area, the b-scan showed a small area of retinal nerve fibre layer thickening. In this area, after 4 weeks, the b scan OCT showed a focal zone of retinal nerve fibre layer thinning (Fig. 1). At the same time, the visual complain was completely resolved.

## Baseline and follow up of the inner nuclear layers thinning

The OCTA scan showed a capillary drop out of the superficial capillary plexus and the corresponding b-scan showed a round hyporeflective grey dot (optical empty) corresponding to the dark grey spot on the enface view at the level of the retinal whitening area.

**Fig. 2:**
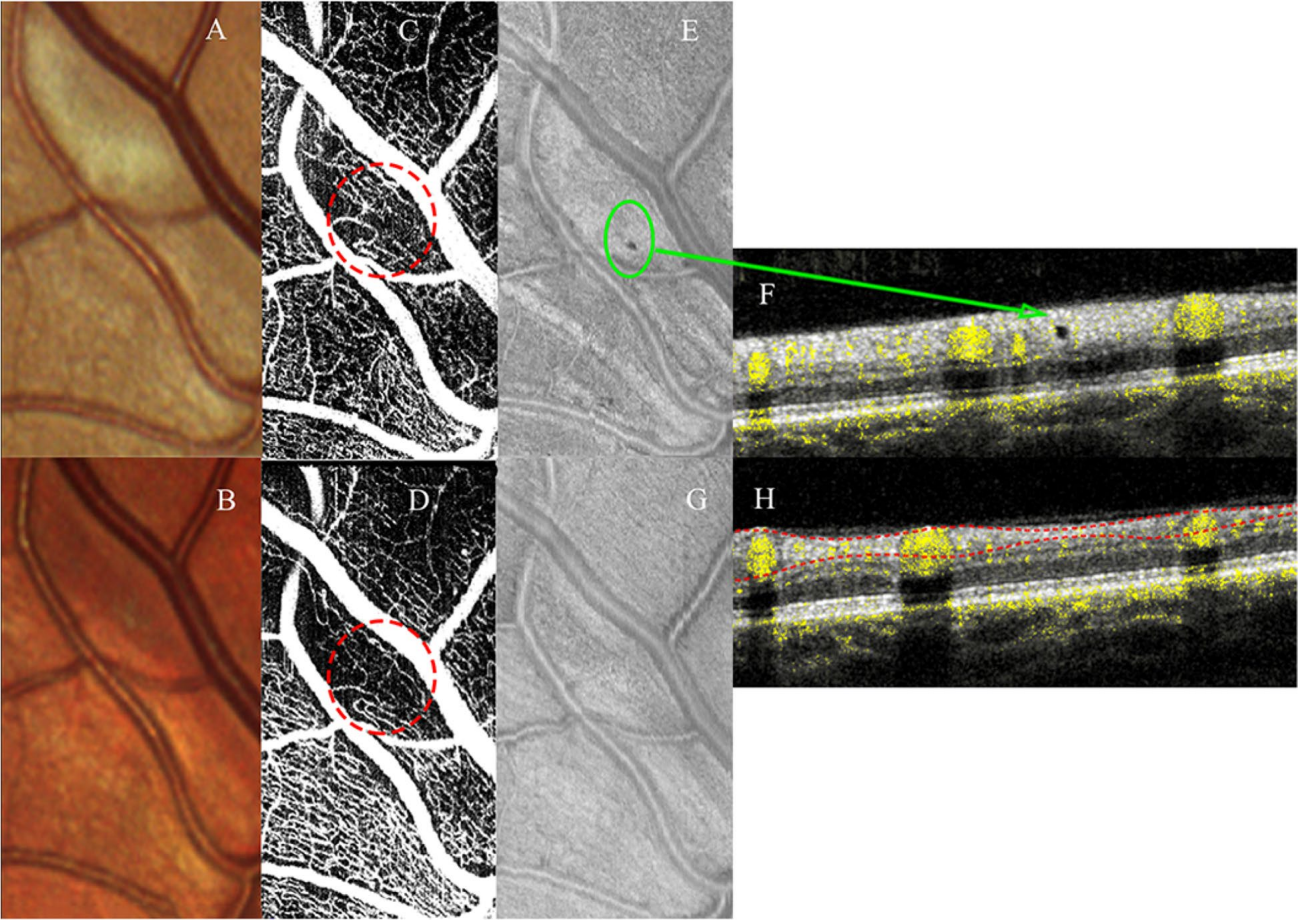
Portion of the colour fundus image of the right eye revealing one retinal whitish lesion (**A**), which was resolved after 4 weeks (**B**). The optical coherence tomography angiography (**C**) showed a capillary drop out at the level of the superficial capillary plexus, which was partially recovered at 4 weeks (**D**). At baseline, on the enface slab (**E**), the greyish area, encompassed between the two large vessel, displayed the dark dot (green circle). On the structural B-scan (**F**), this corresponded to the small hyporeflective dot within the hyperreflectivity band in the inner retina layers. At the follow up visit, in the corresponding area, only a thinning of the retinal nerve fibre layer is present (**G** and **H**)

At follow- up, after 4 weeks, the OCTA scan showed a partial recovery of this focal vessel shut down of the superficial capillary plexus indicating an incomplete restoration of blood flow. The hyporeflective grey dot corresponding to the dark grey spot on the enface slab disappeared. Also, the ophthalmoscopy showed that the focal area of retinal whitening was resolved. (Fig. 2).

## Baseline and follow up of the optical coherence tomography angiography analysis

An echocardiogram and carotid Doppler sonography were obtained to assess for possible embolic retinitis. While the first examination was unremarkable, the second revealed < 50% stenosis within the right carotid bulb primarily indicating the presence of a cholesterol atherosclerotic plaque.

## Discussion and conclusions

Branch artery occlusion can represent the earliest sign of cardiovascular diseases. In the acute phase, the non-perfused or partially perfused retinal area appears white.

It has been already found that the localization of the hyperreflectivity on SD-OCT b-scan can help not only to visualize which retinal layer are involved but also to show the progressive thinning of respective layers the retinal damage with time [6, 7].

This patient who complained of blurred vision in her right eye, with consistent focal OCTA findings, was diagnosed with branch retinal artery occlusion, which can be both associated with either retinal emboli or vasospasm. Very few cases have been described in the literature to date, with limited details regarding the patient history and follow-up.

Today, optical coherence tomography angiography provides the ophthalmologists with the new capability to promptly observe and assess the retinal changes due to the vascular impairment over time.

In patients with severe Raynaud's phenomenon, Salmenson et al. described persistent decrease in retinal capillary flow after cold stimuli, resolved at least 10 min after removal [8]. Furthermore, Ansari Y et showed the OCTA findings in the superficial capillary plexus in a case of sudden onset of peripheral visual deficit secondary to retinal artery spasm in Raynaud’s phenomenon [5]. However, the case presented did not suffer with Raynaud's disease.

On the other hand, small microvasculature thromboembolic events of the capillary network in turn can also cause localized ischemic lesions, limited to particular retinal layers.

In a previous study, the appearance of the thromboembolic plaque, based on their component including collagen I, IV and calcium or cholesteryl esters, showed different emission spectrum [9]; nevertheless, OCTA cases of retinal thromboembolic plaque has not been shown.

In addition, since it is known that there is a threshold of the OCTA system necessary to register blood flow rate [10], it is interesting to speculate, as we shown in a previous study on hyporeflective microaneurysm [11], that the blood flow inside the hyporeflective dot might be turbulent or containing plasma with erythrocytes packed, and therefore not shown using OCTA [12, 13].

Moreover, the contribution of the long-term effects of higher dietary protein intake cannot be totally excluded. A previous study showed the relationship between high protein intake and increased incidence of cardiovascular events [14]. Furthermore, it was found that the prevalence of the metabolic syndrome is higher in a diet with low in carbohydrate and high in protein [15].

Finally, in this case reported, the retinal finding could be interpreted as a microcavity/cyst.

The microvacuolar changes have been already described in the macular area in patients with neurodegenerative diseases including autosomal dominant optic atrophy, multiple sclerosis, ischemic optic neuropathy, glaucoma [16–19]. This represents the result of swelling from diminished fluid clearance of the inner retina rather than from retinal vessels exudation. Müller cells have been implicated in fluid absorption and their dysfunction might be correlated with formation of micro-lacunae also in case with branch retinal artery occlusion.

In case with branch retinal artery occlusion, the OCTA imaging might help to identify in vivo and to caught in the act the specific region of the retinal impairment.

However, fundus autofluorescence and fluorescein angiography were not performed in this case and this can be considered a limitation of this study. Therefore, further studies are needed to discern between vasospasm as well as thromboembolic plaques, including the differentiation of their component, but also, the possible formation of small microcavity leading to new insights into the pathophysiology of this retinal disease.

## Data Availability

All data generated or analysed during this study are included in this published article.

## Abbreviations

OCTA: Optical coherence tomography angiography
OCT: Optical coherence tomography
